# Factors associated with the time to treat breast cancer in the pandemic period: an observational study

**DOI:** 10.1590/0034-7167-2022-0428

**Published:** 2023-08-07

**Authors:** Denise Montenegro da Silva, Régia Christina Moura Barbosa Castro, Ana Fátima Carvalho Fernandes, Erilaine de Freitas Corpes, Cristina Poliana Rolim Saraiva dos Santos, Andrea Bezerra Rodrigues

**Affiliations:** I Universidade Federal do Ceará. Fortaleza, Ceará, Brazil

**Keywords:** Breast Neoplasms, Outpatient Care, Comprehensive Health Care, Nursing Care, Covid-19., Neoplasias de la Mama, Atención Ambulatoria, Atención Integral de Salud, Atención de Enfermería, COVID-19., Neoplasias da Mama, Assistência Ambulatorial, Assistência Integral à, Saúde, Cuidados de Enfermagem, Covid-19.

## Abstract

**Objectives:**

to analyze the factors associated with the time to surgical treatment for breast cancer in patients seen at a reference mastology outpatient clinic in the State of Ceará.

**Methods:**

analytical, longitudinal study with medical charts from the Mastology Outpatient Clinic of Assis Chateaubriand Maternity School. We used 140 medical charts of breast cancer patients with surgeries performed during the pandemic.

**Results:**

the study evidenced associations between schooling and shorter time to treatment in patients who underwent biopsy before the first outpatient visit (p = 0.026; OR: 0.16; CI = 0.03-0.85); in the group who had the biopsy performed by the outpatient clinic, was associated the type of tumor (p = 0.019) and neoadjuvant therapy (p = 0.000).

**Conclusions:**

the lesser educational level, tumor type, and use of neoadjuvant therapy were factors associated with the time to treatment during the pandemic period.

## RESUMO

**Objetivos:**

analisar os fatores associados ao tempo para tratamento cirúrgico do câncer de mama em pacientes atendidas em ambulatório de mastologia de referência no estado do Ceará.

**Métodos:**

estudo analítico, longitudinal, realizado com prontuários do Ambulatório de Mastologia da Maternidade Escola Assis Chateaubriand. Foram utilizados 140 prontuários de pacientes com câncer de mama com cirurgias realizadas durante o período pandêmico.

**Resultados:**

o estudo evidenciou associações entre a escolaridade e o menor tempo para tratamento nas pacientes que realizaram biópsia antes da primeira consulta ambulatorial (p = 0,026; OR: 0,16; IC = 0,03-0,85); no grupo que realizou a biópsia pelo ambulatório, estiveram associados o tipo de tumor (p = 0,019) e a neoadjuvância (p = 0,000).

**Conclusões:**

o menor nível educacional, o tipo de tumor e a utilização da neoadjuvância foram fatores associados ao tempo para tratamento durante o período pandêmico.

## RESUMEN

**Objetivos:**

analizar los factores relacionados al tiempo para tratamiento quirúrgico del cáncer de mama en pacientes atendidas en ambulatorio de mastología de referencia en el estado de Ceará.

**Métodos:**

estudio analítico, longitudinal, realizado con prontuarios del Ambulatorio de Mastología de la Maternidad Escuela Assis Chateaubriand. Fueron utilizados 140 prontuarios de pacientes con cáncer de mama con cirugías realizadas durante el período pandémico.

**Resultados:**

el estudio evidenció relaciones entre la escolaridad y el menor tiempo para tratamiento en las pacientes que realizaron biopsia antes de la primera consulta ambulatoria (p = 0,026; OR: 0,16; IC = 0,03-0,85); en el grupo que realizó la biopsia por el ambulatorio, estuvieron relacionados el tipo de tumor (p = 0,019) y la neoadyuvancia (p = 0,000).

**Conclusiones:**

el menor nivel educacional, el tipo de tumor y la utilización de la neoadyuvancia fueron factores relacionados al tiempo para tratamiento durante el período pandémico.

## INTRODUCTION

The pandemic caused by the SARS-Cov-2 virus, which causes the disease known as covid 19, soon showed a high potential for transmissibility, alerting health agencies to elaborate and implement actions to reduce transmission^(1)^.

The Brazilian government adopted mandatory biosafety measures, such as personal protection equipment, hand washing, isolation of cases and contacts, and social distancing, among many others^(2)^. Numerous health services needed to be readapted since the demand for hospital beds was increasing, especially those in intensive care.

Due to changes in priorities in health services, the care of cancer patients has become a dilemma since health professionals need to redesign oncologic assistance^(3)^. Regarding breast cancer, the pandemic situation implied the interruption or change of treatments, delay in surgeries and consultations, affecting the quality of life of these patients^(4)^.

Studies guide the best forms of treatment for patients with breast cancer to be adopted during the covid-19 pandemic; among them was the significant increase in patients undergoing oral therapy, implementation of online consultations, and reduction of chemotherapy and surgical procedures^(3)^.

Many health institutions opted to reduce surgeries, postponing reconstruction surgeries and those for low-grade malignant breast tumors^(5-6)^. The priority of cases was for those presenting advanced staging or those in which not performing the procedure could result in poor prognoses, such as inflammatory breast cancer and triple-negative breast cancer after completion of neoadjuvant chemotherapy^(7-8)^.

The timely access of those patients to the healthcare network influences survival since one of the most critical factors of this pathology is the time elapsed between diagnosis and the beginning of treatment. Thus, this study intended to evaluate the factors that influenced the time to surgical treatment of women with breast cancer during the covid-19 pandemic.

## OBJECTIVES

To analyze the factors associated with the time to treatment for breast cancer in patients seen at a reference mastology clinic in the State of Ceará (CE).

## METHODS

### Ethical aspects

The project was submitted and approved by the Research Ethics Committee (CEPE) of the Assis Chateaubriand Maternity School, which belongs to the Federal University of Ceará.

### Design, period and place of study

This article results from a dissertation with an analytical and retrospective design, conducted with data from medical charts from the Mastology Outpatient Clinic of the Assis Chateaubriand Maternity School. That and the HUWC constitute the Federal University of Ceará Hospital Complex. The study site was chosen, in particular, because it is a reference service in caring for women with breast cancer, especially for oncologic breast surgeries. Data collection occurred from November 2021 to January 2022.

### Population and sample

The population of this study was women diagnosed with breast cancer who attended the mastology outpatient clinic from March 2020 to July 2021. We included charts of women diagnosed with breast cancer regardless of stage, positive biopsy, with surgical treatment modality of choice. The women’s medical charts in palliative care at the end of life were excluded from the trial due to the belief that this moment is appropriate for therapies other than oncology.

The expected sample was estimated considering a confidence interval of 95% (expressed in standard deviation as 1.96) and a maximum error of 5%. The population size was related to the number of surgeries. According to the management reports on care production^(9)^, the mastology medical team performed an average of 328 surgeries in women with breast cancer per year in the last five years.

After applying the finite sample, it reached the desired value of 174 women. From March 2020 to July 2021, 189 new breast cancer cases with elective surgery were registered. Of these, 11 presented patients in palliative care, and 38 for insufficient information, such as the date of biopsy and/or others related to the periods investigated. Due to the reduction in the number of surgeries because of the pandemic and the application of exclusion criteria, it was collected 140 medical charts at the end of the year.

### Study protocol

Initially, we went to the outpatient clinic to consult the registry book; afterward, a spreadsheet was created with the patient’s name, chart number, type of surgery, and telephone number. After this step, with the authorization of the service nurse, the University Hospitals Management Application (AGHU) was accessed, promoting the search in the listed medical charts and data collection. At another time, we passed those data to the analysis program.

We obtained clinical aspects, medical exams, and sociodemographic data through a direct physical search and/or electronic medical charts in two stages. A semi-structured form Collected Information about: a) Sociodemographic data: Age, Race, Marital Status, Schooling, Income, Religion, Origin, Profession; b) Reproductive history: Pregnancies, Parities, Abortions, Menarche, Menopausal status; c) Social habits: Alcohol and tobacco consumption at diagnosis; d) Comorbidities: Hypertension, Diabetes mellitus, Other comorbidities; e) Clinical-laboratory data: Histological type of tumor, Clinical TNM staging system, Immunohistochemical markers, First implemented treatment, Sentinel lymph node biopsy, Time between diagnosis by biopsy and first treatment (Surgery/QT/Hormone).

At first, the patients were divided into two groups, according to the place request and diagnosis biopsy. Those with a biopsy diagnosis verified before the first visit to the outpatient clinic were allocated to the Before Referral (PR) group, and who came to the clinic without a defined diagnosis and whose request and diagnosis were verified after the first visit to the referral, were allocated to the Biopsy by Referral (BR) group.

As a cutoff point, the value referred to in Federal Law 12,732 was used to evaluate the time of initial treatment and verify the behavior of those during the pandemic. This law establishes that cancer patients have the right to start treatment (whether chemotherapy, radiotherapy, or surgery) within 60 days from the pathology report confirming the diagnosis of the disease. Thus, it was a delay when the time between the first diagnosis of cancer and the beginning of treatment was more than 60 days.

### Analysis of results and statistics

Data were tabulated in a Microsoft Office Excel 2010 spreadsheet and later stored and processed by the Statistical Packpage for the Social Sciences (SPSS), version 23.0. They were analyzed according to descriptive statistics, by measures of major tendency, dispersion, and normality tests for continuous variables.

The chi-square test and Fisher’s exact test were applied to verify the differences in proportions. The times between diagnosis and first treatment were dichotomized at the cutoff point of 60 days and configured as dependent variables. Sociodemographic variables were analyzed to verify associations. The p-value < 0.05 indicated statistical significance. The results were presented in tables and discussed in light of the pertinent literature, presenting the Odds Ratio (OR) and considering the 95% confidence interval.

## RESULTS

From the total sample, regarding sociodemographic characteristics, the age of patients ranged from 26 to 85 years old, prevailing the age range of 50 to 69 years old, with 57 (47.9%) women. As for the other variables, 57 (43.8%) had no level of education, 91 (66.5%) had a partner, 89 (67.9%) were catholic, 72 (51.8%) came from the capital Fortaleza, 70 (52.2%) said they had no profession. The most prevalent comorbidities were systemic arterial hypertension (SAH) in 53 (37.8%); and diabetes mellitus (DM) in 33 (23.6%).

In the groups’ division, it was observed that in the PR group, 48 women were submitted to biopsy before their first visit to the outpatient clinic, since in the BR group, 92 were submitted to biopsy at the request of the ACMS.

It was associations of the times of diagnosis by biopsy and the first treatment implemented (considering neoadjuvant as the first treatment) with the sociodemographic variables, tumor type, neoadjuvant therapy, type of surgery, and oncoplastic technique (Table 1 and Table 2).

**Table 1:** Association of variables with the time of diagnosis by biopsy before the first visit to the referral and with the first treatment of patients with surgical treatment, Fortaleza, Ceará, Brazil, 2022

Variable	Time to treatment - PR group				
	General	< 60 days	> 60 days	OR	*p* value
Age at diagnosis					0.229
< 50	23 (47.9)	9 (18.8)	14 (29.2)		
50-69	20 (41.7)	6 (12.5)	14 (29.2)		
> 70	5 (10.4)	0 (0.0)	5 (10.4)		
Patient origin				1.77 (0.49-6.43)	0.504
Capital	15 (31.3)	6 (12.5)	9 (18.8)		
Interior/Others	33 (68.8)	9 (18.8)	24 (50.0)		
Years of study				0.16 (0.03-0.85)	0.026
< 9	17 (37.0)	2 (4.3)	15 (32.6)		
> 9	29 (63.0)	13 (28.3)	16 (34.8)		
Partner				1.03 (0.26-4.08)	0.965
Yes	35 (72.9)	11 (22.9)	24 (50.0)		
No	13 (27.1)	4 (8.3)	9 (18.8)		
Income source				0.72 (0.20-2.48)	
Yes	24 (52.2)	7 (15.2)	17 (37.0)		
No	22 (47.8)	8 (17.4)	14 (30.4)		
Type of tumor					0.193
IDC	31 (64.6)	12 (25.0)	19 (39.6)		
DCIS	5 (10.4)	0 (0.0)	5 (10.4)		
Others	12 (25.0)	3 (6.3)	9 (18.8)		
Neoadjuvant				3.04 (0.85-10.86)	0.083
Yes	17 (35.4)	8 (16.7)	9 (18.8)		
No	31 (64.6)	7 (14.6)	24 (50.0)		
Type of surgery				3.07 (0.85-11.07)	0.080
Mastectomy	23 (47.9)	10 (20.8)	13 (27.1)		
Quadrantectomy	25 (52.1)	5 (10.4)	20 (41.7)		
Oncoplastic technique				2.33 (0.65-8.31)	0.186
Yes	16 (33.3)	7 (14.6)	9 (18.8)		
No	32 (66.7)	8 (16.7)	24 (50.0)		

*PR - Before Referral; IDC - Invasive Ductal Carcinoma Ductal; DCIS - Ductal Carcinoma In Situ.*

**Table 2:** Association of variables with the time of diagnosis by biopsy by referral and with the first treatment of patients with surgical treatment, Fortaleza, Ceará, Brazil, 2022

Variable	Time to treatment - BR group				
	General	< 60 days	> 60 days	OR	*p* value
Age at diagnosis					0.128
< 50	30 (32.6)	23 (25.0)	7 (7.6)		
50-69	47 (51.1)	26 (28.3)	21 (22.8)		
> 70	15 (16.3)	8 (8.7)	7 (7.6)		
Patient Origin				1.20 (0.50-2.86)	0.824
Capital	57 (62.6)	36 (39.6)	21 (23.1)		
Interior/Outros	34 (37.4)	20 (22.0)	14 (15.4)		
Years of study				1.15 (0.48-2.77)	0.749
< 9	40 (47.6)	25 (29.8)	15 (17.9)		
> 9	44 (52.4)	26 (31.0)	18 (21.4)		
Partner				0.82 (0.33-1.99)	0.661
Yes	56 (62.9)	33 (37.1)	23 (25.8)		
No	33 (37.1)	21 (23.6)	12 (13.5)		
Income source				0.82 (0.34-1.95)	0.658
Yes	40 (45.5)	24 (27.3)	16 (18.2)		
No	48 (54.5)	31 (35.2)	17 (19.3)		
Type of tumor					0.019
IDC	51 (55.4)	34 (37.0)	17 (18.5)		
CDIS	16 (17.4)	5 (5.4)	11 (12.0)		
Others	25 (27.2)	18 (19.6)	7 (7.6)		
Neoadjuvant				4.91 (1.98-12.15)	0.000
Yes	53 (57.6)	41 (44.6)	12 (13.0)		
No	39 (42.4)	16 (17.4)	23 (25.0)		
Type of surgery				1.75 (0.77-4.11)	0.218
Mastectomy	39 (42.4)	27 (29.3)	12 (13.0)		
Quadrantectomy	53(57.6)	30 (32.6)	23 (25.0)		
Oncoplastic technique				2.05 (0.72-5.84)	0.173
Yes	23 (25.0)	17 (18.5)	6 (6.5)		
No	69 (75.0)	40 (43.5)	29 (31.5)		

*CDI - Carcinoma Ductal Invasivo; CDIS - Carcinoma Ductal In Situ.*

The study showed associations between the PR group and schooling (p = 0.026; OR: 0.16; CI = 0.03-0.85): women who were referred with diagnosis by biopsy before the first consultation and higher level of education (≥ nine years) had 84% less chance in the time to late treatment, configuring this factor as protective. The PR group was composed mainly of women from the interior and/or other states (68.8%), and the time between biopsy and treatment was more significant than 60 days for 68.7% of them. For this group, neoadjuvant therapy was low, used in 31 (64.6%) of them.

As for the BR group, the period counted from diagnosis by a biopsy performed by the outpatient clinic until treatment was associated with tumor type (p = 0.019) and neoadjuvant therapy (p = 0.000), showing that patients who underwent neoadjuvant therapy were 4.91 times more likely to receive treatment in the shortest time. This group was composed mainly of women from the capital who received treatment in less than 60 days, 58 (63.0%) of them.

## DISCUSSION

The low level of education, the type of tumor, and the use of neoadjuvant therapy were factors associated with the time to treatment during the pandemic. This data reinforces the health determinants in the opportune search for specialized services and corroborates the importance of health professionals by emphasizing clinical reasoning in managing patients in particular situations.

It is widely accepted that the social context in which people live, and work influences their health perception. Levels of education levels have been related to the incidence of breast cancer, stage at diagnosis, and survival due to a better understanding of health perception and early search for services^(10)^.

The health services amid the pandemic needed remodeling to meet the new demands of the population. In several cases, reduced access to hospitals and operating rooms were imposed, implying the establishment of priorities in the care of cancer patients^(11-12)^.

There is an increasing recognition that the care of patients with breast cancer will depend on highly individualized clinical characteristics. In this regard, healthcare professionals excel at integrating the multidisciplinary team in developing a practical treatment approach that considers the stage of tumor presentation, the biological subset of breast cancer, and the genetic factors underlying breast cancer risk^(13)^.

The tumor-focused treatment personalization and use of neoadjuvant therapy allowed optimization in the initiation of therapy, which was crucial during the pandemic and presented unique challenges in the care of cancer patients. International consensus emphasized neoadjuvant therapy as the preferred initial treatment approach, capable of providing effective systemic treatment (equivalent to adjuvant therapy) to prevent cancer recurrence, and especially indicated when there was any suggestion that the response to treatment might allow the de-escalation of surgery or radiotherapy^(13-14)^.

Infiltrating ductal carcinoma is one of the most common diagnostic types of breast cancer and one of the most common histologic types found in the advanced stages of the disease. As this tumor is highly aggressive, it is understandable that it is associated with a shorter delay in treatment compared to other histologic types, as reported in studies^(15)^.

In the current study, patients referred from other health services presented, in the majority, a longer time for treatment. Moreover, social characteristics, such as the patient origin and schooling, point to social inequalities experienced in accessing and using health services.

The use of health services is complex, resulting from the interaction of several factors, among which we can mention health needs, the characteristics of services and professionals, the geographical accessibility, among others^(16-17)^. In the context of a pandemic, such inequalities accentuate due to the actions to contain the spread of the virus.

The quickly advancing covid-19 pandemic, the declaration of all hospitals as pandemic hospitals, social isolation, especially in the elderly and people with comorbidities, coupled with anxiety around the disease have led to the delay in diagnosis and treatment of breast cancer, as hospitalizations of both patients with complaints and patients with a routine screen have decreased^(18-19)^.

The interval between diagnosis and initiation of treatment, in particular, has been an object of concern in several countries. In Brazil, aligned with public policies concerning the right of cancer patients, Law No. 12,732 came into effect on November 22, 2012, establishing the right of cancer patients to begin chemotherapy, radiotherapy, or surgical treatment within 60 days of the signed pathology report diagnosing the disease.

Studies show that delays of up to 60 days supporting the validity of that measure do not negatively affect oncological outcomes, especially in early-stage breast cancers, regardless of the biological tumor^(20-21)^.

In China, a study found a mean time of 58.55 ± 24.87 to treatment days during the outbreak stage of the pandemic. At the beginning of the pandemic, authorities’ temporarily halted transportation between cities and provinces to prevent the virus from spreading, which led to expected delays in diagnosis and treatment. In addition, nothing was known about the virus at the beginning of the pandemic, and fear of covid-19 caused many to voluntarily avoid social contact, which may have been the main reason for the increased time to treatment^(22)^.

In Pakistan, a study conducted in the Breast Surgery Section of the tertiary care cancer unit found no difference in the time to treatment in the groups investigated between the pre-covid-19 and covid-19 generations. This result is similar to that evidenced in another study conducted in the Canadian province of British Columbia, in which no significant difference in surgical care was shown compared to the pre-covid-19 era, with better delay time, which they associated with a low impact of covid-19 in their center and better care based on the multidisciplinary team’s performance^(23)^.

In the Brazilian context, a cohort study conducted in São Paulo showed decreased breast exams and diagnoses only in the first 90 days of the pandemic, with a partial compensation in diagnoses in the second period, although a 35% reduction in the number of mammograms performed in the year before the pandemic^(24)^.

Furthermore, an ecological study conducted with data from 2016 to 2020 demonstrated a significant drop in mammograms performed during the pandemic throughout Brazil, negatively impacting screening and early diagnosis^(25)^.

In this context, it must optimize the diagnosis and treatment of cancer patients, and managing these patients needs adaptation to the best available resources. The need for any interventional procedure must be balanced against the increased risk during a pandemic and evaluated on a case-by-case basis to address the urgency of the procedure and the effect on the patient outcome when delaying the procedure^(26)^.

Overall, the management of breast cancer underwent considerable changes as a response to covid-19, which varied according to geographic area and pandemic phase and between different institutions within a given country. Professional bodies have recommended alternative therapeutic options as a short-term imperative^(13,27)^.

A trend of broadening the indications for primary systemic therapy was evident with the increasing severity of the pandemic and pressure on emergency services. The American Society of Clinical Oncology (ASCO) reported an increase in neoadjuvant chemotherapy in response to a decrease in first surgeries^(28)^. In the current study, there was an association between a shorter treatment time and the neoadjuvant therapy.

### Study limitations

Limitations include the small sample size and the single-institution cohort design. However, these findings represent essential data for assessing the impact of covid-19 on breast cancer care until more important registry data become available.

### Contributions to the fields, Nursing, Health or Public Policy

Access to health services for early diagnosis and timely initiation of treatment for breast cancer is still a problem since the covid-19 pandemic continues to impact health and the economy on several continents. Therefore, knowing the behaviors established during the pandemic through studies such as this allows for identifying health needs and forming clinical reasoning to implement the best conduct, directly influencing the organization and assistance to provide for these patients.

The data found reinforces, even if indirectly, the importance of the participation of the multidisciplinary team in its performance when performing the clinical reasoning for the best patient conduct in the context of a pandemic.

## CONCLUSIONS

In summary, our findings show that patients referred with diagnoses from other health services and a lesser education level experienced longer delays concerning breast cancer treatment. Patients diagnosed by the outpatient clinic had shorter periods between diagnosis and treatment associated with tumor type and use of neoadjuvant therapy.

Screening protocols and treatments that direct professional performance in unforeseen scenarios are essential. Thus, it is necessary to continue creating programs to improve technical and scientific knowledge so that healthcare professionals can keep themselves constantly updated for better care management.
